# Assessing the burden of Scorpionism: Epidemiological trends and health outcomes in Northwest of Iran

**DOI:** 10.1371/journal.pntd.0013201

**Published:** 2025-07-03

**Authors:** Madineh Abbasi, Mehran Shahi, Hossein Barahoei, Ahmad Ali Hanafi-Bojd, Ali Banagozar Mohammadi, Mahasti Alizadeh, Mostafa Farmani, Simin Khayatzadeh, Karim Gerami, Abdollah Badzohreh, Aida Amirijavid, Saideh Yousefi

**Affiliations:** 1 Infectious and Tropical Diseases Research Center, Sina hospital, Tabriz University of Medical Sciences, Tabriz, Iran; 2 Department of Medical Entomology & Vector Control, School of Public Health, Infectious and Tropical Diseases Research Center, Hormozgan Health Institute, Hormozgan University of Medical Sciences, Bandar Abbas, Iran; 3 Agriculture Institute, Research Institute of Zabol, Zabol, Iran; 4 Department of Vector Biology & Control of Diseases, School of Public Health, Tehran University of Medical Sciences, Tehran, Iran; 5 Zoonoses Research Center, Tehran University of Medical Sciences, Tehran, Iran; 6 Internal Medicine Department, Faculty of Medicine, Tabriz University of Medical Sciences, Tabriz, Iran; 7 Medical Philosophy and History Research Center, Tabriz University of Medical Sciences, Tabriz, Iran; 8 Province Health Center, Tabriz University of Medical Sciences, Tabriz, Iran; 9 Department of Disease Prevention and Control, Health Deputy, Maragheh University of Medical Sciences, Maragheh, Iran; 10 Department of Nursing and Midwifery, Sara. C., Islamic Azad University, Sarab, Iran; 11 Education Organization of Sarab, Sarab, Iran; 12 Sirjan School of Medical Sciences, Sirjan, Iran; 13 Student Research Committee, Sirjan School of Medical Sciences, Sirjan, Iran; Institut Pasteur de Tunis, TUNISIA

## Abstract

**Background:**

While only a limited number of scorpion species are classified as dangerous to humans, the potentially life-threatening effects of their stings classify scorpionism as a global health concern. Iran, with its high scorpion diversity, reported more than 63,000 scorpion sting cases in 2023. This study aims to elucidate the epidemiological characteristics of scorpion envenomation in northwest Iran.

**Methods:**

This retrospective descriptive cross-sectional study was conducted over a period of two years (2022–2023) in northwest Iran. The research focused on scorpion sting cases that required treatment at 25 scorpion sting treatment centers (SSTCs) across the East Azerbaijan Province. Data were collected from scorpion sting cases presenting for treatment. Statistical analyses were performed, using Chi² and Mann-Whitney tests for both descriptive and analytical evaluations. Geographic distribution maps were generated to illustrate the locations of sting incidents relative to treatment facilities.

**Result:**

During two years, 3,154 scorpion sting cases were reported in East Azerbaijan Province, Iran. Most patients were aged 31 to 40 years, with 54.9% being male. Most stings occurred in urban areas (48.7%) and primarily indoors (75%). Remarkably, 99.96% of cases resulted in full recovery, with only one death reported. Treatment methods included wound cleaning (50.8%) and the administration of antivenom (53.2%). The results indicate scorpion stings peak during the summer months, with the highest frequency occurring between midnight and 2 AM.

**Conclusion:**

This study highlights the public health challenge posed by scorpion stings in East Azerbaijan Province. While recovery rates are high, further efforts are needed to improve public health interventions, including educational programs for vulnerable groups such as farmers and children. Enhancing access to medical care and timely treatment is essential to reducing morbidity and mortality. Future research should focus on local scorpion species and develop tailored prevention strategies to mitigate scorpionism.

## Author summary

Scorpion stings present a significant public health challenge in many regions, particularly in northwest Iran, where scorpions diversity is high. This study investigates the epidemiological trends and health outcomes associated with scorpion envenomation in East Azerbaijan Province over a two-years (2022–2023). We analyzed data from 3,154 reported cases, focusing on demographics, treatment modalities, and recovery rates. Our findings reveal that the majority of victims are males; specifically, those aged 31 to 40, with peak sting incidents occurring during the summer months, predominantly indoors. Remarkably, 99.96% of cases resulted in full recovery, highlighting the effective treatment protocols. However, one death was documented. The data emphasize the need for targeted public health interventions, including educational programs targeting vulnerable groups such as farmers and children. Enhancing community awareness, as well as ensuring access to timely medical care, can significantly mitigate the impact of scorpionism in affected populations. Future research should explore the specific scorpion species present in the region and develop tailored prevention strategies to further reduce the burden of this neglected tropical disease.

## Introduction

Scorpions are among the oldest arthropods [1] and have significant medical importance due to their venomous nature [2]. For many years, they have attracted humans attebtions due to their unique morphology, painful stings, mortality rate, and behavioral characteristics [1]. Only a limited number of scorpion species are considered dangerous to humans; however, the potentially life-threatening effects of a single sting are significant enough to classify scorpionism as a global health concern [3]. While scorpion stings are reported across all continents (excluding Antarctica), the epidemiology of scorpionism remains inadequately understood [4].

The incidence and severity of scorpion sting envenomation are higher in seven global regions, including the Northern Sahara, southern and eastern Africa, the Middle East, South India, Mexico, Brazil, and the Amazon basin [3,5]. Scorpion stings are the most important cause of arachnid envenomation in many tropical and subtropical regions, with approximately 1.5 million cases are reported annually and nearly 3,000 deaths worldwide [6,7]. In the Middle Eastern and North African regions (MENA), scorpion stings account for 42% of the global sting burden, with about half of the related mortalities [8]. Iran, Saudi Arabia, Algeria, Egypt, Israel, Morocco, and Tunisia Countries are particularly affected by scorpionism [8].

Globally, approximately, 2,540 species of scorpions belonging to 21 families have been described, but only a small percentage of them are potentially dangerous to human [9,10]. The Middle East is known as one of the regions with the high diversity of scorpions [11]. This remarkable diversity appears to result from evolutionary adaptations to the arid climate conditions frequently found in the area [11]. The most medically important scorpion species in MENA region are belonging to the Butidae family [12]. In the Middle East, 13 species from the Butidae family including *Androctonus amorous*, *Androctonus australis*, *Androctonus bicolor*, *Androctonus crassicauda*, *Hottentota jayakari*, *Hottentota saulcyi*, *Hottentota schach*, *Hottentota zagrosensis*, Leirus *jordanensis*, *Leirus hebraeus*, *Leirus quinquestriatus*, *Mesobuthus caucasicus*, *Mesobuthus eupeus,* as well as species from the Hemiscorpiidae and Scorpionidae families including *Hemiscorpius acanthocercus*, *Hemiscorpius lepturus*, and *Nebo hierochonticus* are considered medically important species, respectively [12].

Iran is known for its extensive scorpion diversity, including several medically significant species. The country hosts four families of scorpions, encompassing 91 species across 20 genera, 61 of which are endemic [13,14]. In 2023, there were 63,760 reported cases of scorpion stings in Iran, with six death [15]. The Buthidae is the most dominant family in Iran, followed by Hemiscorpiidae and Scorpionidae families [16]. The sting agents in Iran comprise 12 species, among which *M. eupeus*, *Compsobuthus matthiesseni*, *H. saulcyi*, *Odontobuthus* doriae, *H. lepturus*, *Orthochirus scrobiculosus*, and *A. crassicauda* are the most medically relevant [17].

East Azerbaijan Province, located in the northwest of Iran, has a low richness of dangerous scorpions due to the climatic conditions. Up to now, seven species of scorpions have been recorded in this province, including *A. crassicauda, M. caucasicus*, *M. eupeus*, *H. saulcyi*, *Mesobuthus vesiculatus*, *O. doriae*, and *Compsobuthus petriolii* of which the first four species are recognized as medically important [9,12]. It seems that the most prevalent species in this province is *M. eupeus*, which has been identified as the main scorpion sting agent [17,18]. The lethal dose (LD_50_) of scorpion venom in dangerous species is less than 1.5  mg/kg in mice; for *M. eupeus* this value is 1.45 mg/kg, making it less dangerous compared to *A. crassicauda* (LD_50_ = 0.08-0.50 mg/kg) [17]. However, studies on scorpions in the region are limited, and more research is needed to identify medically important species in the area.

Scorpion stings are often overlooked as a tropical disease in various areas, highlighting the need for greater awareness and enhanced medical care to decrease illness and death rates [19]. The epidemiology of scorpion envenomation epidemiology reveals critical patterns essential for understanding its public health implications. Various studies indicate that the incidence of scorpion stings is influenced by geographic, climatic, and socioeconomic factors [20,21]. Additionally, demographic factors, including age and occupation, play a significant role in the susceptibility to scorpion stings, with children and agricultural workers bing particularly vulnerable [22,23]. Understanding these epidemiological trends is vital for developing effective prevention strategies and targeted interventions to reduce both the incidence and severity of scorpion stings in affected populations.

Scorpion envenomation can lead to a range of clinical symptoms, which vary in severity depending on the species involved, the dose of venom injected, and the individual’s response to the venom. This response is influenced by various factors including the patient’s overall health and past medical history. however, common symptoms include localized pain, swelling, and redness at the sting site in mild cases of envenomation. In severe cases, localized necrosis can occur, along with systemic effects such as neurological disturbances, respiratory difficulties, cardiovascular complications, and coagulation disorders [24]. The variability in clinical presentation highlights the importance of prompt medical attention and appropriate treatment protocols to mitigate the potentially life-threatening consequences of severe envenomation [25].

Management of scorpion stings is crucial, as timely intervention can significantly reduce morbidity and mortality rates. Effective treatment strategies often involve the administration of analgesics, antivenom, and supportive care tailored to each patient’s clinical status [26]. Furthermore, integrating epidemiological data into public health initiatives is essential for designing and implementing effective prevention and treatment programs. Educating communities about the risks associated with scorpion stings and the importance of promotly seeking medical help can enhance patient outcomes and reduce fatalities [27]. By analyzing epidemiological factors, healthcare providers can better prepare for scorpion envenomation cases and develop targeted strategies that address the needs of at-risk populations [28,20].

In East Azerbaijan Province, northwest Iran, there is a notable lack of information on scorpion envenomation. Previous research has primarily focused on southern provinces, neglecting the northern and colder regions, such as East Azerbaijan, which is among the coldest area in the northwest Iran. Given that agriculture is the predominant occupation in this province, villagers are particularly vulnerable due to their close interaction with the soil and natural environments.

However, the abundance of dangerous species can vary in neighboring areas, which affects the rates of scorpionism and mortality  [18]. Understanding the epidemiology of scorpionism is essential for developing effective prevention strategies and targeted interventions to reduce the incidence and severity of scorpion stings in affected populations. Due to the lack of comprehensive studies on the epidemiology of scorpionism and identification of high-risk places, times, and occupations in the study area, this study aims to elucidate the epidemiological characteristics of scorpion envenomation in East Azerbaijan Province. By investigating scorpion stings in this region, the study seeks to inform prevention and treatment strategies, thereby enhancing community awareness and safety.

## Materials and methods

### Ethics statement

This study was conducted with ethical approval from the regional ethics committee of Tabriz University of Medical Sciences, under code: IR.TBZMED.AEC.1402.080 (approval date: 11/27/2023).

### Study design and location

This study is a descriptive retrospective, cross-sectional analyzis conducted over a two-year period (2022–2023) in East Azerbaijan Province, northwest Iran. This province has three medical universities: Tabriz University of Medical Sciences (covering 19 counties), Maragheh University of Medical Sciences, and Sarab University of Medical Sciences (both serving one county). The study targeted scorpion sting cases that received treatment at 25 scorpion sting treatment centers (SSTCs) in hospitals and health centers throughout the province.

### Data collection

The study included all individuals who presented for treatment of suspected scorpion stings at SSTCs, according to the Iran CDC’s scorpion sting management guidelines, as outlined by the Iranian Ministry of Health and Medical Education [29].

The inclusion criteria were patients who presented with a history of a recent scorpion sting, either witnessed by the patient or a caregiver, or suspected based on clinical manifestations consistent with scorpion envenomation [29]. The study did not exclude any patients based on the availability of scorpion specimen for identification. Both confirmed and suspected scorpion sting cases were included.

The Iranian Ministry of Health maintains an online system where data for each scorpion sting case presented to SSTCs are recorded. This system includes demographic information (age, gender), the region of residence, month, season, time of sting (day vs. night), site of sting on the body, scorpion species, and timing intervals between the sting and reaching the hospital (in hours) as well as the antivenin injection (hours). The identification of the scorpion species was primarily based on the patients’ descriptions of the color of the scorpion involved in the sting. If the patient reported a black scorpion, it was classified as *A. crassicauda*, and if a yellow scorpion was mentioned, other species such as *M. eupeus* or *O. doriae*, which are commonly reported in the province, were considered [9,12].

The scorpion antivenom used for patient treatment is ScoFab, an equine-derived polyvalent F(ab’)2 antivenom manufactured by Padra Serum Alborz in Iran. ScoFab is indicated for treating patients envenomated by six scorpion species commonly found in the region: *A. crassicauda, Buthotus saulcyi, Buthotus schach, O. dorae, M. eupeus, and H. lepturus* [29].

### Treatment protocols

All treatments provided to patients and symptoms observed at the time of admission were categorized into two groups: local and systemic symptoms [29]. According to the national protocol for scorpion sting management in Iran, antivenom may be administered based on clinical presentation without requiring prior species identification for both confirmed and suspected cases. Treatment of scorpion sting patients depends on multiple factors, including the type of scorpion, time of sting (day vs. night), location of the sting, patient age, the time elapsed before patient reached the hospital, and the presence of systemic symptoms [29].

#### • Statistical analysis.

Data were analyzed using SPSS version 27 for both descriptive and analytical statistical analyzes, using Chi², the Kruskal-Wallis H test and Spearman’s rank correlation with a confidence level of 95%. Daily admission rates for scorpion stings were calculated monthly to assess the variations in incidence throughout the year. The locations of the patients’ bites were identified using geographical coordinates obtained from Google Maps. The map was created using ArcMap version 10.7, with base layers sourced from OpenStreetMap.

## Results

**Fig 1:**
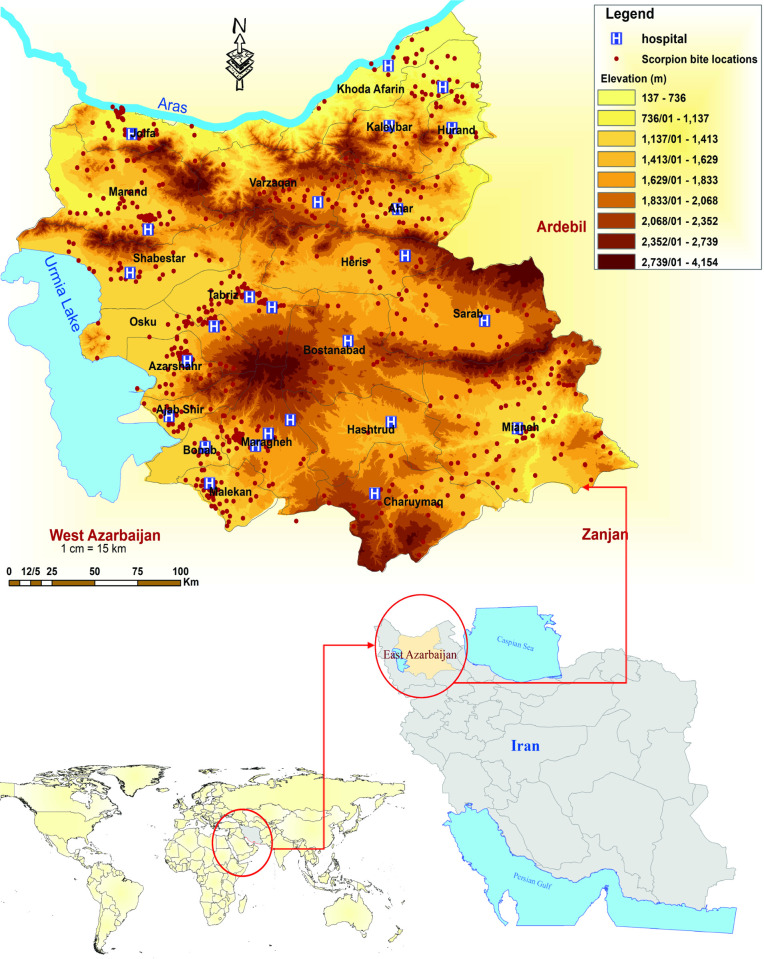
Map of the study area and distribution of scorpion sting cases and treatment centers in East Azerbaijan Province, northwest Iran, 2022–2023. Basic map data is available under the Open Database License (ODbL). Reference: https://www.openstreetmap.org.

A total of 3,154 scorpion stings were reported, with Tabriz University of Medical Sciences covering area whit the highest frequency of scorpion stings at 88.5%, followed by Maragheh (9.2%) and Sarab (2.3%) Universities of Medical Sciences. Among the 25 SSTCs, Sina Hospital in Tabriz had the highest number of admissions for scorpion sting patients by treating 804 cases (25.5%), and followed by Imam Khomeini Hospital in Osku County, with 285 cases (9.0%). Fig 1, illustrates the geographical locations of all patients who visited the SSTCs. The analyzis of the elevation at scorpion sting locations showed an elevation range from 175 to 2,702 m, with a mean elevation of 1,378.54 ± 318.32 m above sea level (Fig 1).

**Fig 2:**
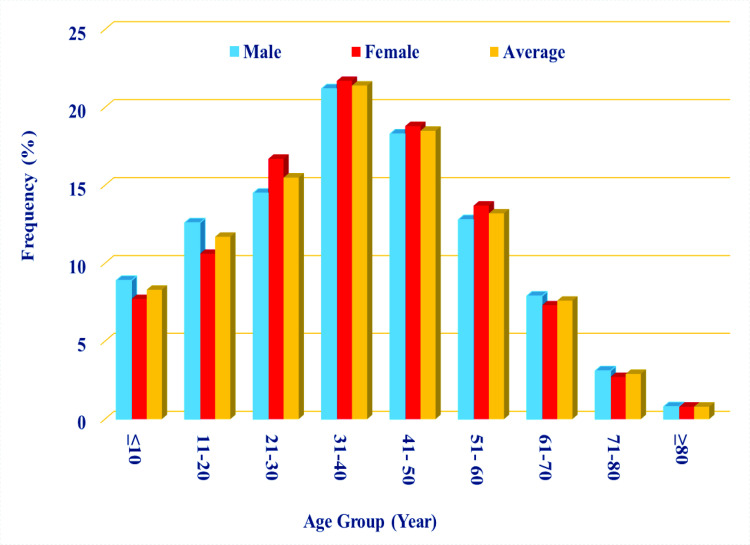
Frequency of scorpion sting by age group and gender, East Azerbaijan Province, northwest Iran, 2022-2023.

The ages of the patients ranged from 1 to 96 years, with a mean age of 36.4 ± 15.2 years. The majority of scorpion stings occurred in individuals aged 31 to 40 (21.4%), with males comprising 54.9% of total incidents (Table 1). The Pearson Chi² analyzis indicated no statistically significant association between age groups and gender (Chi² = 7.999, p-value = 0.434) (Fig 2).

**Table 1:** Environmental and demographic factors associated with scorpion sting in East-Azerbaijan Province, northwest Iran, 2022-2023.

Category		Frequency	
		N.	%
**Sting area**	**Village**	1560	49.4
	**City**	1537	48.7
	**Natural landscape**	57	1.8
**Sting location**	**Indoor**	2365	75
	**Outdoor**	789	25
**Occupation**	**Child (unemployed)**	162	5.1
	**Student**	586	18.6
	**Farmer**	1535	48.7
	**Household**	144	4.6
	**Self-employed**	727	23.1
**Anatomical site**	**Hand/Arm**	1572	49.8
	**Leg/Foot**	1136	36.0
	**Trunk**	331	10.5
	**Head and neck**	115	3.6
**Education level**	**Illiterate**	451	14.3
	**High school diploma**	2463	78.1
	**Master’s degree**	240	7.6
**Underlying health conditions**	**No**	3053	96.8
	**Yes**	101	3.2
**Total**		**3154**	**100**

**Fig 3:**
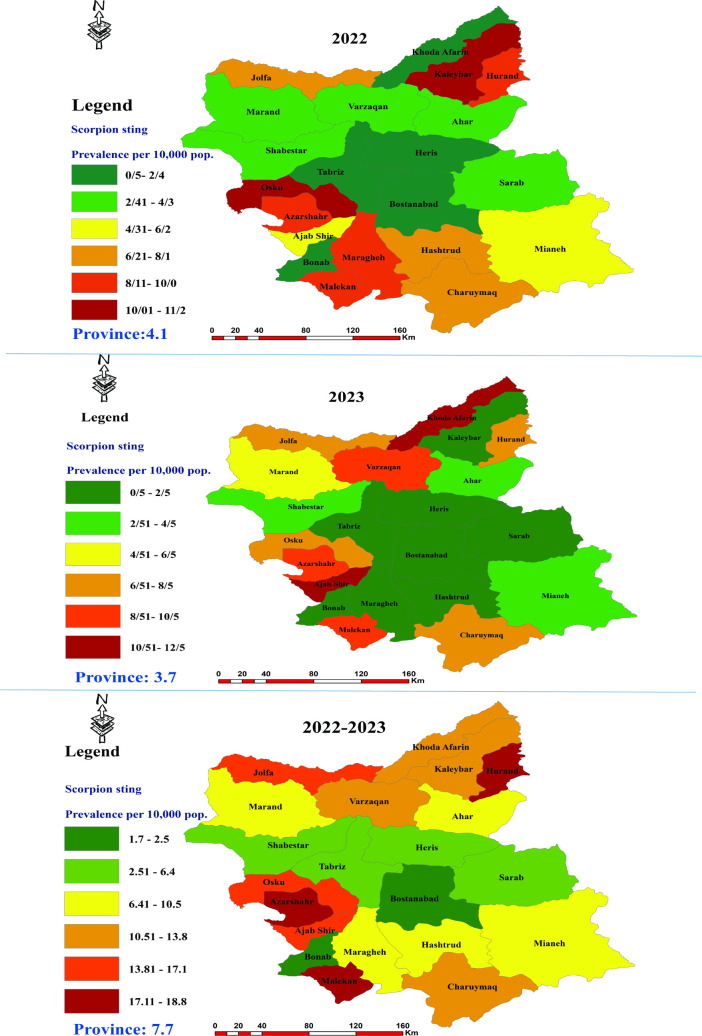
Geospatial distribution of scorpion sting incidence across Counties of East Azerbaijan Province, northwest Iran, 2022–2023, Map data is available under the Open Database License (ODbL). Reference: https://www.openstreetmap.org.

The overall incidence rates of scorpion stings in East Azerbaijan Province were 4.1, 3.6 and 7.8 cases per 10,000 population in 2022, 2023, and over the study period, respectively. At the county level, Kaleybar reported the highest incidence in 2022 (11.9 cases per 10,000 population), while Ajab Shir had the highest incidence in 2023 (12.7 cases per 10,000 population). Conversely, Bonab (2022) and Bostan Abad (2023) reported the lowest incidence of 0.5 per 10,000 population in both years. Fig 3 illustrates the incidence of scorpion stings in the studied years through color-coding, effectively categorizing the data by county for each year (Fig 3). For details on the frequency of scorpion sting cases by district in East Azerbaijan Province, please refer to S1 Table.

**Fig 4:**
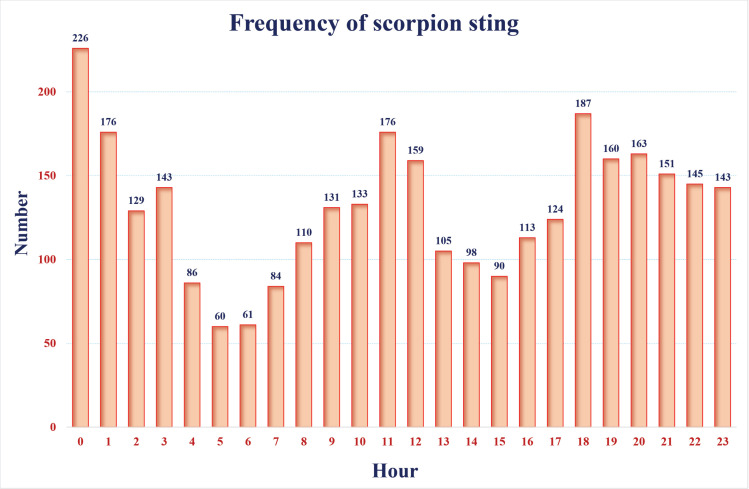
Scorpion stings frequency over 24 hours in East Azerbaijan Province, northwest Iran, 2022–2023.

The analyzis of scorpion sting frequency over 24-hour period revealed three prominent peaks: the first peak occurred in midnight; the second and third peaks were ocurred in 11:00 AM and  18:00, respectively (Fig 4).

**Fig 5:**
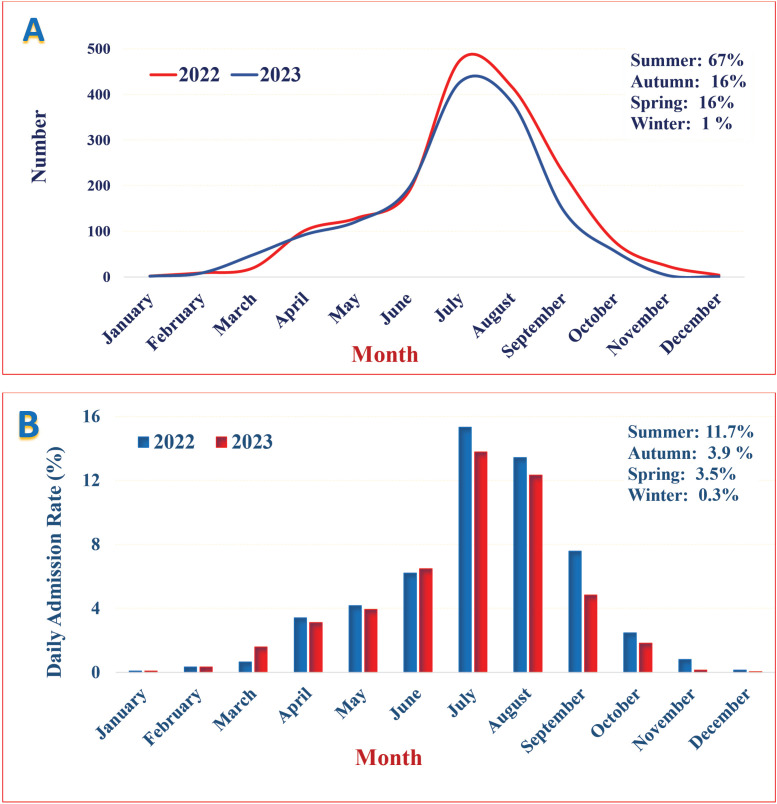
The status of scorpion stings throughout the year in East Azerbaijan Province, northwest Iran, 2022-2023; Categorized by number (A) and Daily Admission Rate (B) in different months.

The analyzis of scorpion stings throughout the year, categorized by month, highlighted a significant increase in cases during July and August. Notably, peak occurrences were observed during the summer, with approximately 67% of scorpion sting incidents occurring, followed by 16% in autumn and spring and 1% in winter. (Fig 5A). The Kruskal-Wallis H test results indicated a significant difference among scorpion stings across different months (p-value<0.001). The daily admission rates for scorpion stings demonstrate a similar trend, with approximately 15.32 cases per day in July and 0.13 cases per day in December. The average daily admission rates are as follows: 11.7 in summer, 3.50 in spring, 3.28 in autumn, and 0.32 in winter (Fig 5B).

The scorpion stings occur in urban (48.7%) and rural areas (49.4%) and are nearly equally represented, the majority occur indoors (75%). The anatomical distribution of scorpion stings shows a higher frequency on the hands and arms (49.8%). Demographically, most individuals affected are farmers (48.7%) whithin occupational groups, and high school graduates (78.1%) in education levels, with a striking majority reporting no underlying health condition (96.8%). Table 1 details the environmental and demographic factors of scorpion stings (Table 1).

The findings indicate that 99.96% of scorpion sting cases resulted in recovery, with only one case (0.04%) leading to death in a 5-year-old child from Jolfa County, who was injured by an *Androctonus* scorpion. Treatment methods included wound cleaning (50.8%), wound dressing (26.6%), analgesics (100%), antivenom (53.2%), antihistamines (36.0%), antibiotics (10.7%), corticosteroids (32.0%), tetanus vaccine (10.1%) and crystalloids (7.8%) (Table 2).

**Table 2:** Status of symptoms/treatment/outcomes of scorpionism in East-Azerbaijan Province, northwest Iran, 2022–2023.

Category	Subcategory	Frequency	
		N.	%
Class I: Local symptoms	Pain and burning	3152	99.9
	Itching	63	2.0
	Ecchymosis	733	23.2
	Swelling	733	23.2
	Local paraesthesia	289	9.1
	Erythema	340	10.7
	Numbness, tingling	300	9.5
Class II: Minor symptoms	Headache	60	1.9
	Low blood pressure	20	0.6
	Tachycardia	253	8.0
	High blood pressure	173	5.5
	Muscle cramps	733	23.2
	Nausea	316	10.0
	Vomiting	14	0.4
	Miosis	733	23.2
Class III: Severe symptoms	Systemic: Restlessness – sgitation – selirium – snconsciousness and seizures	43	1.4
	Sympathetic nervous system stimulation: mydriasis and dry mouth	142	4.5
	Parasympathetic nervous system stimulation: increased salivation, tearing, urination, and Incontinence and frequency	253	8.0
Treatment category	Wound cleaning	1601	50.8
	Wound dressing	839	26.6
	Analgesics	3153	100
	Antivenom	1679	53.2
	Antihistamines	1134	36.0
	Antibiotics	337	10.7
	Corticosteroids	1010	32.0
	Tetanus vaccine	317	10.1
	Crystalloids	246	7.8
Outcome	Recovery	3153	99.96
	Death	1	0.04

**Fig 6:**
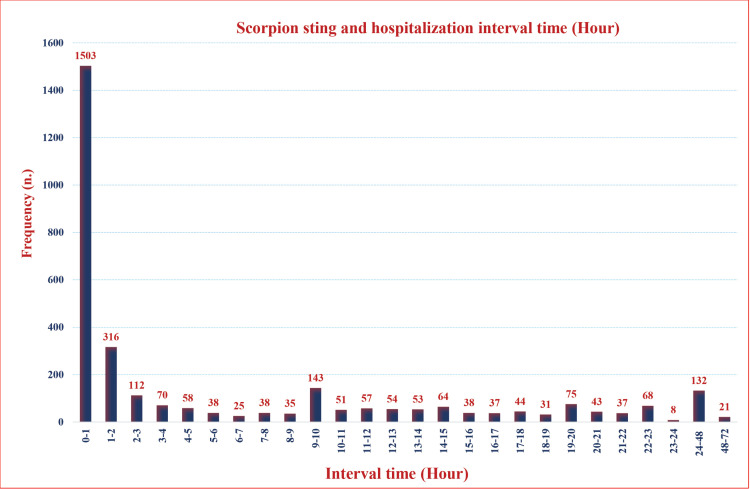
Hospitalization timeline following scorpion stings in East Azerbaijan Province, northwest Iran, 2022-2023.

Regarding local symptoms, pain and burning were the predominant symptoms reported in 3,152 cases (99.9%), with ecchymosis and swelling occurring in 733 cases (23.2%). Other minor symptoms, such as headache (1.9%) and tachycardia (8.0%), were also observed, while severe complications were documented in 14% of cases (Table 2). A total of 2,124 doses of antivenom have been administered over two years. Fig 6 depicts the number of patients who received hospital admission after a scorpion sting, categorized by the time elapsed since the sting. The highest number of visits, 1,503 patients, occurred within the first hour following the sting. Following this initial peak, the number of visits gradually decreased over the subsequent time intervals. This declining trend suggests that most patients received medical attention in the early hours following the sting, and the number of hospital admissions decreased over time (Fig 6). S2 Table shows detailed information about the relationship between hospitalization time post-scorpion sting and clinical symptoms (S2 Table).

## Discussion

Scorpionism is a significant public health issue globally and meets all the criteria for recognition as a neglected tropical disease (NTD). Approximately 2.5 billion people are at risk of scorpion stings, and about 1.5 million scorpion stings, are reported annually [5,19,30–32]. However, the actual number of scorpionism cases is likely higher, as many cases go unrecorded in health systems for various reasons [33]. This underreporting highlights the need for better surveillance systems to accurately capture the incidence of scorpionism to inform public health strategies.

Studies have shown that the average incidence rates of scorpion stings varies across different regions of Iran [34–38]. In this study area, the incidence rate varies among different regions and the marginal areas of the province, especially the bordering counties on Urmia Lake, located in West Azerbaijan Province, exhibiting a higher incidence than other areas. A study conducted in West Azerbaijan Province showed that the cases of scorpionism in counties neighbouring East Azerbaijan, which is our study area, are high [33]. This finding suggests that targeted public health interventions are necessary in these high-risk areas to reduce scorpion stings and their consequences.

In the present study, men accounted for 55% of the victims, with most of cases detected in the age group of 31–40 years, followed by the 41–50 age group. The gender distribution corroborate findings from other studies conducted in the northwest, south, and central regions of Iran, as well as in countries such as Turkey, Iraq, Saudi Arabia, Jordan, India, and Brazil [11,33,39–46]. However, these findings contrast with finding from studies conducted in the southeast, southwest, and some areas of southern Iran, as well as Brazil [31,47–50]. It seems that the differences in scorpionism between genders may be due to factors such as the types of patients’ occupations, scorpion species behaviour (endophilic or exophilic), and the higher propensity of women to seek medical care in some cultures [49,51]. This highlights the importance of developing gender-specific educational programs that address the unique risks faced by men and women in these communities. The age group can be a risk factor for scorpionism, and the higher incidence of scorpion stings among youth may bedue to their outdoor occupations [52]. In studies conducted in the south, southeast, and northwest of Iran, as well as in some regions of India and Brazil, individuals over the age of 20 presented more scorpion stings [31,33,42,45,47,48,53]. There is a different classification of age groups in studies conducted in various areas, so we cannot compare our results with studies conducted in Kashan County and Sistan and Baluchistan, Qom, and Khuzestan Provinces of Iran, as well as Saudi Arabia and India [31,40,48,52,54].

Scorpion stings are events that depend on ecological and environmental factors such as temperature and rainfall patterns can influence scorpion behaviour, subsequently increasing or decreasing the incidence of scorpionism [38,55]. In the present study, the number of scorpionism cases varied among different months, with the maximum number of patients observed during hot months. This seasonal variation has underscored the need for public health campaigns during peak months to educate at-risk populations about preventive measures. The findings of his investigation agree with numerous studies conducted in various regions of Iran, as well as in Brazil, Morocco, India, Iraq, and Oman [23,33,35,38,43,45,48,51,56–60]. But, these findings do not align with findings from studies conducted in some areas of Brazil, which report no significant difference in scorpionism prevalence among the months [51,61,62]. Scorpions undergo hibernation, and their activity is influenced by mean temperature, so, in areas with slight temperature variation, these creatures are remine active throughout the year, while in areas with seasonal temperature changes, their activity is limited to certain months [47].

Generally, infrastructure problems such as poor housing conditions, lack of basic sanitation due to unplanned urban development, and the specific ecological conditions of rural areas provide an ideal habitat for scorpions, leading to scorpion stings [47,51]. The results of correlation between patients’ living places and the prevalence of scorpionism vary; for example, in many studies conducted in the northwest, southwest, south, and central parts of Iran, as well as in Brazil and Sudan, the majority of scorpion sting cases occurred in urban areas [23,33,34,39,40,51,63,64]. In contrast, in some studies conducted in the southwest and south of Iran, Turkey, Saudi Arabia, and India, the highest cases were reported from rural regions [38,47,65–69]. In our study, the incidence of scorpionism was approximately equal in rural (49.4%) and urban areas (48.7%). This indicates that public health strategies must equally address urban and rural populations, ensuring that preventive measures and resources are available in both settings. Most stings occurred indoors, with an outdoor-to- indoors sting ratio of 25:75, indicating that human habitats are high-risk environments for scorpion stings in the study area. These results are consistent with findinds from studies conducted in the northwest, southwest, southeast, and central parts of Iran, as well as in Brazil [45,49,51,53,54,70].

Some species of scorpions are adapted to human dwellings and come out of their shelter at night and sting humans indoors. However, some species do not enter human habitats and instead hide under stones, in underground burrows, and under debris outdoors [51,71,72]. Although scorpions are nocturnal, contact during daytime work or recreation can also result in stings [73]. Therefore, in a recent study, cases of scorpionism occurring during the day were also observed. In terms of occupation, it should be noted that, most cases happen to farmers in both urban and rural areas, and these people are exposed to scorpion stings at home and at work. Educational programs should focus on this job groups.

Scorpions are nocturnal arthropods that hide in the crevices of dwellings, under stone, in underground burrows, and under debris during the day and coming out of their shelter at night [71,72]. Therefore, in the present study, although scorpion stings are reported at all hours of the day, most cases occur in the first hours of the morning (between 0–2 AM) which is similar to the study conducted in the northwest of Iran [45] and contrary to the studies conducted in the south of Iran and Brazil which showed that the most scorpion sting occurred at the first half of the night and during the day, respectively [47,73].

In this study, hands were the most affected body site, corresponding to 50% of all scorpion sting cases. This finding coincides with previous studies conducted in Sistan and Baluchistan, Qom, Hormozgan, and Fars Provinces [40,42,47,48]. In Iran, hands and feet are generally stung more frequently than other parts of the body [40,48,65,74–76]. However, several activities, such as farm-related work and doing activities in the dark, increase the risk of scorpion stings [72].

Various factors influence clinical outcomes in patients, including those related to the agent (scorpion species, size of scorpion, depth of sting, number of stings, season, and venom composition), the host (age, body weight, physiological condition, underlying diseases, sting site, and history of previous sting), and the socio-economic environment (access to health services and presence of trained personnel in medical centers) [58,77–79]. In our study, Pain with burning was the most common symptom among victims, with NSAIDs (nonsteroidal anti-inflammatory drugs), steroids, and antihistamines being the alternative drugs used by 32% and 36% of patients, respectively. 53.2% of patients received antivenom, and the mortality rate was 0.04%, observed in one child under 5 years old who received antivenom. The mortality rates in southeast and northwest Iran were 0.03 and 0.07, respectively, while in Iraq, it was zero [33,43,48]. In our study area, similar to findings from a study conducted in India, a high percentage of patients were managed with symptomatic and supportive measures and did not receive antivenom. However, in some studies, all patients were treated with antivenom [31,44,67].

Generally, in some studies conducted in various parts of Iran about 85% of scorpion sting cases received antivenom [33,42]. As mentioned above, various factors are involved in the clinical outcomes in patients. According to Mahadevan et al., scorpions can regulate venom injection; therefore, in some cases, a sting may occur without venom being injected [72]. This is one of the main reasons for the absence of severe symptoms observed in patients without antivenom. Although scorpion antivenom is widely used in many countries and some physicians believe that, it is the most effective treatment for scorpion envenoming, But, studies conducted in India and Tunisia suggest that its role in managing scorpion stings is remains debatable [31,80]. Therefore, it is crucial to conduct further research into the effectiveness of antivenom treatments in different regions and to evaluate alternative management strategies. Therefore, in addition to providing health education for at-risk groups to prevent scorpion stings and promote early medical intervention, it is recommended that further research be conducted by relevant experts to evaluate the effectiveness of antivenom treatment. These studies should consider the predominant scorpion species present in each region, as variations in venom composition may influence the clinical outcomes and optimal management strategies for envenomation. Additionally, enhancing health education for at-risk groups about prevention and early medical intervention can significantly reduce the impact of scorpionism.

## Conclusion

This study underscores the significant public health challenge posed by scorpion stings in East Azerbaijan Province, Iran, with reported over 3,000 reported cases in two years, particularly affecting agricultural communities. Most victims are males aged 31 to 40, with a higher incidence during summer months and occurring predominantly indoors. While recovery rates are high, the need for improved public health interventions is critical for reducing the impact of scorpion stings. Future research should focus on comprehensive epidemiological studies to monitor treatment outcomes and evaluate intervention effectiveness. Targeted educational programs for vulnerable populations and enhanced access to timely medical care, including the appropriate use of antivenom, are essential for reducing morbidity and mortality associated with scorpion stings. Addressing these factors will help mitigate the burden of scorpionism and improve health outcomes for affected communities.

### Limitation

This study is limited to cases of scorpion stings that sought treatment at healthcare facilities that provide scorpion sting treatment services. Consequently, the findings may not represent the total incidence of scorpion stings in the province. Several factors contribute to these limitations. Firstly, individuals with mild symptoms may choose not to seek medical attention, resulting in an under-representation of such cases. Secondly, also, the distance from treatment centers might stop some patients from getting care, which could affect the data. Additionally, the collected data is based on the information registration forms provided by the Ministry of Health, which may not encompass all relevant clinical information. As a result, certain treatment cases could be overlooked due to omissions in these forms.

Furthermore, it is important to note that the identification of scorpions was primarily based on patient descriptions, which may lead to inaccuracies, especially in cases where identification was not possible, such as stings occurring during night-time or when scorpions were not brought for identification. These limitations highlight the necessity for further research to better understand the full scope of scorpion sting incidents in the region.

## Supporting information

S1 Table Frequency of scorpion sting by counties in East Azerbaijan Province, northwest Iran, 2022–2023. (DOCX)

S2 Table Relationship between hospitalization time post-scorpion sting and clinical symptoms in East Azerbaijan Province, northwest Iran, 2022–2023. (DOCX)

S3 Table Scorpion sting incidence rates per 10,000 population by counties in East Azerbaijan Province, northwest Iran, 2022–2023. (XLSX)

S4 Table Hourly frequency of scorpion stings in East Azerbaijan Province, northwest Iran, 2022–2023. (XLSX)

S5 Table Age and gender demographics of scorpion sting cases in East Azerbaijan Province, northwest Iran, 2022–2023. (XLSX)

S6 Table Duration of hospitalization post-scorpion sting in East Azerbaijan Province, northwest Iran, 2022–2023. (XLSX)

S7 Table Location coordinates of scorpion stings in East Azerbaijan Province, northwest Iran, 2022–2023 (Duplicate coordinates have been removed to enhance clarity and prevent overlap on the map). (XLSX)
