# Supplementary material for: Assessing the burden of Scorpionism: Epidemiological trends and health outcomes in Northwest of Iran
Source: PLoS Negl Trop Dis. 2025 Jul 3;19(7):e0013201. doi: 10.1371/journal.pntd.0013201 (PMC12286322; doi:10.1371/journal.pntd.0013201)
Supplement: S1 Table — (DOCX) [file pntd.0013201.s001.docx]

**Supplementary 1.** Frequency of scorpion sting by counties in East Azerbaijan Province, Northwest of Iran, 2022-2023

| County | Year | | Total N. (%) |
| --- | --- | --- | --- |
|  | **2022 N. (%)** | **2023 N. (%)** |  |
| Tabriz | 405 (24.3) | 440 (29.6) | 845 (26.8) |
| Maragheh | 270 (16.2) | 21 (1.4) | 291 (9.2) |
| Osku | 166 (9.9) | 116 (7.8) | 282 (8.9) |
| Marand | 109 (6.5) | 139 (9.4) | 248 (7.9) |
| Azar Shahr | 105 (6.3) | 109 (7.3) | 214 (6.8) |
| Malekan | 108 (6.5) | 104 (7.0) | 212 (6.7) |
| Miyaneh | 82 (4.9) | 82 (5.5) | 164 (5.2) |
| Ajab Shir | 32 (1.9) | 93 (6.3) | 125 (4.0) |
| Ahar | 56 (3.4) | 55 (3.7) | 111 (3.5) |
| Jolfa | 50 (3.0) | 51 (3.4) | 101 (3.2) |
| Shabestar | 46 (2.8) | 44 (3.0) | 90 (2.9) |
| Sarab | 40 (2.4) | 31 (2.1) | 71 (2.3) |
| Varzeqan | 23 (1.4) | 47 (3.2) | 70 (2.2) |
| Kaleybar | 57 (3.4) | 9 (0.6) | 66 (2.1) |
| Hashtrud | 43 (2.6) | 14 (0.9) | 57 (1.8) |
| Charoymaq | 21 (1.3) | 22 (1.5) | 43 (1.4) |
| Khoda Afarin | 4 (0.2) | 37 (2.5) | 41 (1.3) |
| Horand | 20 (1.2) | 18 (1.2) | 38 (1.2) |
| Bonab | 7 (0.4) | 29 (2.0) | 36 (1.1) |
| Heris | 14 (0.8) | 18 (1.2) | 32 (1.0) |
| Bostan Abad | 12 (0.7) | 5 (0.3) | 17 (0.5) |
| Total | **1,670 (100)** | **1,484 (100)** | **3,154 (100)** |
