# Supplementary material for: Assessing the burden of Scorpionism: Epidemiological trends and health outcomes in Northwest of Iran
Source: PLoS Negl Trop Dis. 2025 Jul 3;19(7):e0013201. doi: 10.1371/journal.pntd.0013201 (PMC12286322; doi:10.1371/journal.pntd.0013201)
Supplement: S2 Table — (DOCX) [file pntd.0013201.s002.docx]

**Supplementary 2.** **Relationship between hospitalization time post-scorpion sting and clinical symptoms** **in East-Azerbaijan Province, northwest Iran, 2022-2023**

| **Symptoms** | | **Hospitalization and scorpion sting interval time** | | | | **Total** | **Chi^2^** | **p-value** |
| --- | --- | --- | --- | --- | --- | --- | --- | --- |
|  |  | **<6 hours**  **n. (%)** | **6-12 hours**  **n. (%)** | **12-24 hours n. (%)** | **>24 hours n. (%)** |  |  |  |
| **Antivenom** | Yes | 1106 (71.9%) | 170 (11.1%) | 169 (11.0%) | 93 (6.0%) | 1538 | 94.374 | 0.000* |
|  | No | 991 (61.4%) | 179 (11.1%) | 383 (23.7%) | 61 (3.8%) | 1614 |  |  |
| **Pain at the bite site** | Yes | 2097 (66.6%) | 348 (11.0%) | 551 (17.5%) | 154 (4.9%) | 3150 | 5.374 | 0.146 |
|  | No | 0 (0.0%) | 1 (50.0%) | 1 (50.0%) | 0 (0.0%) | 2 |  |  |
| **Swelling and redness at the bite site** | Yes | 261 (79.1%) | 27 (8.2%) | 34 (10.3%) | 8 (2.4%) | 330 | 27.035 | 0.000* |
|  | No | 1836 (65.1%) | 322 (11.4%) | 518 (18.4%) | 146 (5.2%) | 2822 |  |  |
| **Severe muscle pain** | Yes | 492 (67.1%) | 72 (9.8%) | 130 (17.7%) | 39 (5.3%) | 733 | 1.794 | 0.616 |
|  | No | 1605 (66.3%) | 277 (11.5%) | 422 (17.4%) | 115 (4.8%) | 2419 |  |  |
| **Numbness, tngling, and muscle cramps** | Yes | 166 (55.3%) | 67 (22.3%) | 33 (11.0%) | 34 (11.3%) | 300 | 80.460 | 0.000* |
|  | No | 1931 (67.7%) | 282 (9.9%) | 519 (18.2%) | 120 (4.2%) | 2852 |  |  |
| **Nausea** | Yes | 165 (52.2%) | 65 (20.6%) | 31 (9.8%) | 55 (17.4%) | 316 | 163.991 | 0.000* |
|  | No | 1932 (68.1%) | 284 (10.0%) | 521 (18.4%) | 99 (3.5%) | 2836 |  |  |
| **Itching** | Yes | 41 (65.1%) | 8 (12.7%) | 10 (15.9%) | 4 (6.3%) | 63 | 0.554 | 0.907 |
|  | No | 2056 (66.6%) | 341 (11.0%) | 542 (17.5%) | 150 (4.9%) | 3089 |  |  |
| **Headache** | Yes | 46 (76.7%) | 3 (5.0%) | 10 (16.7%) | 1 (1.7%) | 60 | 4.304 | 0.230 |
|  | No | 2051 (66.3%) | 346 (11.2%) | 542 (17.5%) | 153 (4.9%) | 3092 |  |  |
| **Vomiting** | Yes | 7 (50.0%) | 3 (21.4%) | 2 (14.3%) | 2 (14.3%) | 14 | 4.566 | 0.206 |
|  | No | 2090 (66.6%) | 346 (11.0%) | 550 (17.5%) | 152 (4.8%) | 3138 |  |  |
| **Hypotension** | Yes | 20 (100.0%) | 0 (0.0%) | 0 (0.0%) | 0 (0.0%) | 20 | 10.126 | 0.018* |
|  | No | 2077 (66.3%) | 349 (11.1%) | 552 (17.6%) | 154 (4.9%) | 3132 |  |  |
| **Tachycardia** | Yes | 64 (25.3%) | 99 (39.1%) | 19 (7.5%) | 71 (28.1%) | 253 | 584.049 | 0.000* |
|  | No | 2033 (70.1%) | 250 (8.6%) | 533 (18.4%) | 83 (2.9%) | 2899 |  |  |
| **Hypertension** | Yes | 163 (94.2%) | 5 (2.9%) | 2 (1.2%) | 3 (1.7%) | 173 | 63.849 | 0.000* |
|  | No | 1934 (64.9%) | 344 (11.5%) | 550 (18.5%) | 151 (5.1%) | 2979 |  |  |
| **Systemic symptoms** | Yes | 31 (72.1%) | 2 (4.7%) | 6 (14.0%) | 4 (9.3%) | 43 | 3.882 | 0.274 |
|  | No | 2066 (66.5%) | 347 (11.2%) | 546 (17.6%) | 150 (4.8%) | 3109 |  |  |
| **Symptoms of sympathetic nervous system Stimulation** | Yes | 27 (62.8%) | 2 (4.7%) | 4 (9.3%) | 10 (23.3%) | 43 | 33.504 | 0.000* |
|  | No | 2070 (66.6%) | 347 (11.2%) | 548 (17.6%) | 144 (4.6%) | 3109 |  |  |
| **Symptoms of parasympathetic nervous System Stimulation** | Yes | 132 (93.0%) | 5 (3.5%) | 2 (1.4%) | 3 (2.1%) | 142 | 47.631 | 0.000* |
|  | No | 1965 (65.3%) | 344 (11.4%) | 550 (18.3%) | 151 (5.0%) | 3010 |  |  |

- Statistically significant by spearman regression analyzes
